# Electrochemical and Dry Sand Impact Erosion Studies on Carbon Steel

**DOI:** 10.1038/srep16583

**Published:** 2015-11-12

**Authors:** M. Y. Naz, N. I. Ismail, S. A. Sulaiman, S. Shukrullah

**Affiliations:** 1 Department of Mechanical Engineering, Universiti Teknologi PETRONAS, 32610 Bandar Seri Iskandar, Perak, Malaysia; 2 Department of Petroleum Engineering, Universiti Teknologi PETRONAS, 32610 Seri Iskandar, Perak, Malaysia; 3 Department of Fundamental and Applied Sciences, Universiti Teknologi PETRONAS, 32610 Seri Iskandar, Perak, Malaysia

## Abstract

This study investigated the dry and aqueous erosion of mild steel using electrochemical and dry sand impact techniques. In dry sand impact experiments, mild steel was eroded with 45 μm and 150 μm sand particles. Scanning electron microscopy (SEM), energy-dispersive X-ray spectroscopy (EDX) and micro-hardness techniques were used to elaborate the surface morphology of the eroded samples. The results revealed significant change in morphology of the eroded samples. In-depth analysis showed that although the metal erosion due to larger particles was significantly higher, the fines also notably damaged the metal surface. The surface damages were appreciably reduced with decrease in impact angle of the accelerated particles. The maximum damages were observed at an impact angle of 90°. The hardness of the samples treated with 45 μm and 150 μm sand remained in the range of 88.34 to 102.31 VHN and 87.7 to 97.55 VHN, respectively. In electrochemical experiments, a triple electrode probe was added into the metal treatment process. The linear polarization resistance (LPR) measurements were performed in slurries having 5% (by weight) of sand particles. LPR of the samples treated with 45 μm and 150 μm sand slurries was calculated about 949 Ω.cm^2^ and 809 Ω.cm^2^, respectively.

Erosion is generally defined as the material loss from the metal surface that being impacted by a flowing fluid which carries tiny solid particles with a sequence of mechanical actions^1^,^2^. The relation between different definitions of the erosion actually refers to the erosion of metals by various damaging mechanisms. There are many cases where leaking accidents happen due to the erosion of metals caused by coarse sand and fines in the oil and gas industries^2^,^3^. Some cases result in fatality due to leaking of highly pressurized natural gas from the production flow-lines, which may cause explosions as illustrated in Fig. 1.

The oil and gas extracted from the well is inevitably polluted with sand and other solid particles. The unwanted debris is the major source of a number of flow insurance problems in oil and gas industry. Damaging of the fittings and pipelines is also the consequences of the sand impact erosion. If the erosion is not properly predicted, monitored and controlled, it can hamper the entire production process. In some cases, it can cause shut down of the process for an extended period of time. Therefore it is important to understand and predict the sand impact erosion for proper protection of pipelines and equipment against its potential hazards^3^.

The erosion process is further classified into try erosion and aqueous erosion. This study deals with both dry and aqueous erosion processes. The aqueous erosion involves the removal of the material either by bubble collapse or liquid/solid particles impingement. In the present case, the erosion of mild steel in 5% by weight sand slurry and dry sand impact environment was performed with two particle sizes. It is important to mention that the erosion is usually at its extreme in the presence of the sand in the fluid. The slurry or aqueous erosion is also a major issue in hydro-turbines and other fluid machinery where erodent solid particles entrained in the fluid impinging on the metal surface to cause its degradation^3^,^4^.

An electrochemical reaction deteriorates the surface of the material through erosion and corrosion whereas the mechanical force of the solid particles damages the surface through erosion. In an aqueous medium or slurry, the rate of the electrochemical reaction for passive material is relatively lower than the stream flow carrying the solid particles^5^. The flowing fluids can deteriorate the passive layers fully or partly. However, the surface erosion essentially depends on the velocity of the solid particles, impact angle, mechanical strength and sharpness of the particles involved in the process. The breakup of passive films causes the surface depassivation, consequently the rate of electrochemical reaction increases on account of repassivation and the metallic dissolution. However, very shorter impact periods and highly localized deformations may lead to a complicated impact phenomenon^3^,^4^,^5^.

This paper deals with dry and aqueous erosion of mild steel using sand bombardment and electrochemical techniques. The motivation for conducting this work was primarily and interest in quantifying the effect of the sand particle size on the mild steel erosion using two different techniques. It was rarely seen in past reports that the researchers used both techniques at the same time for study of sand impact erosion in production flow-lines. The given work was an effort to fill the existing research gap. The electrochemical tests were conducted using slurry pot erosion mechanism, whereas the dry erosion tests were performed by accelerating and bombarding the sand particles on the steel samples in a dry chamber. The objective of the work was to investigate how the sand erodes the flow-lines. There are several parameters which can affect the erosion of the metal surface such as air velocity, sand flow rate, impact angle, sand weight% in the slurry and distance of the target surface from the nozzle. Herein, effect of the sand particle size, impact angle, weight% and nozzle distance on erosion of the mild steel was investigated through SEM, EDX, LPR measurements and micro-hardness testing.

## Methodology

### Dry Sand Impact Experiments

**Figure 2:**
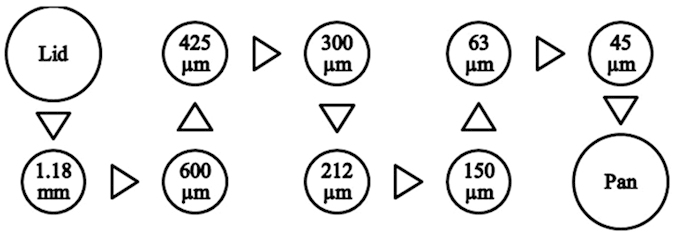
Schematic of the arrangement of the sieves used for dry sand sieving.

Dry sieving method was used to classify the size of the sand particles. These analyses were performed by using a set of sieves with gradually decreasing screen size. Schematic of the arrangement of the sieves used for dry sand sieving is shown in Fig. 2. The dried sand was passed through a stacked set of the sieves of the following mesh sizes: 1.18 mm, 600 μm, 425 μm, 300 μm, 212 μm, 150 μm, 63 μm and 45 μm. The formation sample was placed on the top of the mesh series and it was seeped through the screens until it faced the screen with openings smaller than the grain size. Mechanical vibrations were applied to assist the sand grains in seeping through and on to the various mesh screens. This method was used to obtain coarse and fine sand samples with average size of 45 μm and 150 μm, respectively.

**Figure 3:**
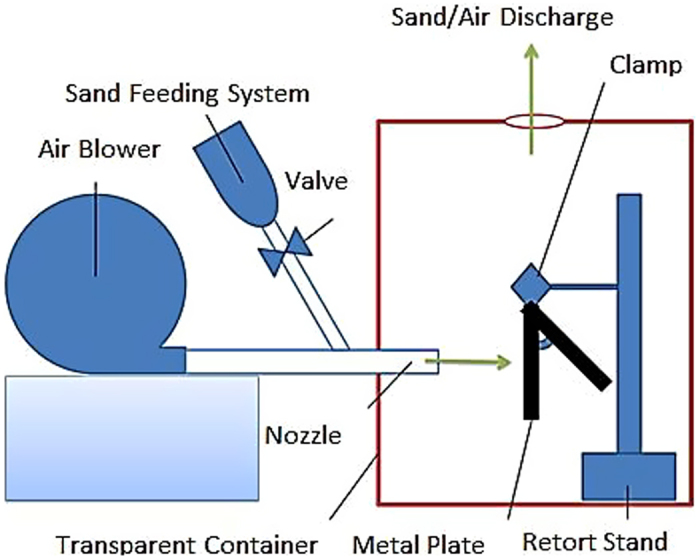
Schematic of the in-house built erosion test rig.

Sand type was sandstone, which is a clastic sedimentary rock of sand-size particles. It was used as an erosion agent for flow-lines erosion. The mild steel plates of 5 mm thickness were bombarded with both coarse and fine sand particles. These plates were representing the inner surface of the production flow-line. The chemical composition of S45C carbon steel was 0.42%–0.48% carbon, 0.15%–0.35% silicon, 0.6%–0.9% manganes, 0.030% maximum phosphorus and 0.035% maximum sulphur. Schematic of the experimental setup for dry sand impact erosion is shown in Fig. 3. A dry sand impact test rig was designed and fabricated to conduct the experiments in air under room temperature (24 °C) and atmospheric pressure (1 atm) conditions. The test section of the rig was a transparent acrylic box. The mild steel samples were mounted at the exit of the nozzle designed in accordance to ASTM guideline. The air-sand mixture was accelerated through a nozzle to a required velocity. The air blower was capable of supplying the air at maximum flux rate of 120 m^3^/h. The air flow velocity was fixed at 20 m/s and steel plates were bombarded with sand at impact angles of 30°, 45°, 60°, 75° and 90°. The sand flow rate was fixed to 1 kg/hr throughout the course of current experimentation. The distance between the steel plate and nozzle was fixed to 10 cm. The surface morphology and composition of the untreated and dry sand treated samples were elaborated using SEM, EDX and Vicker Hardness Number (VHN).

### Electrochemical Experiments

**Figure 4:**
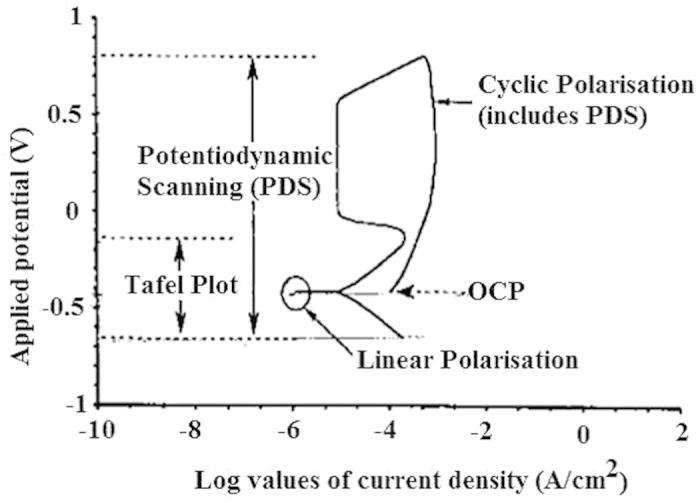
Summary of the possible electrochemical measurements plot.

The electrochemical tests on sand impact erosion-corrosion of the mild steel were conducted using a slurry pot erosion mechanism. A DC technique based on polarization resistance measurements was used to evaluate the surface damaging of the mild steel in the mixtures of tap water and sand with 1%wt sodium chloride (NaCl). The sand content in the slurry was fixed to 5% by weight. The LPR measurement technique was a typical experimental setup composed of a cell with three electrodes. The polarization resistance measurement pathway is summarized in Fig. 4. The LPR measurements were used to obtain *in situ* erosion-corrosion results. A triple electrode system was formed by a specimen as a working electrode, a counter electrode and a reference electrode, and the potentiostat linked to a computer for data capturing. The specimen electrode was polarized and the corresponding current passing between the counter and working electrodes was recorded.

The specimen polarization was controlled by the potentiostat supplying electrons to the working electrode or counter electrode. The electrical neutrality of the electrodes and electrolytes was maintained through the response of the ions to the electrode polarization. Electrochemical active species were also moved to the counter and reacted with electrons supplied by the potentiostat. Herein, the DC polarization of the specimen was based on a change in the potential of the working electrode and measurement of the respective currents produced as a function of potential. The corresponding polarization curves were obtained in the potential range of −20 to +20 mV with a scan rate of 1 mV/min. Using this data, LPR measurements were performed on mild steel in slurries of both 45 μm and 150 μm sand particles. Similar to the dry sand impact erosion tests, the electrochemical experiments were also conducted for a fixed time period of 1 hour.

## Results and Discussion

Over the years, it was believed that the fines in the range of 50–75 μm did not significantly erode the metal due to smaller particle sizes and weak impact on the target surface. However, it has been observed that the fine particles can escape through most of the sand screens, which make them almost inevitable in oil and gas production. These fines may severely damage the installations at location where coarse sand cannot reach in normal situations^4^,^5^. The evidences from the oil and gas industry proved that the sand is one of the major causes of pipeline erosion. In the given work, dry and aqueous sand erosion of the mild steel was investigated for better understating of the sand erosion in the production flow-lines.

**Figure 5:**
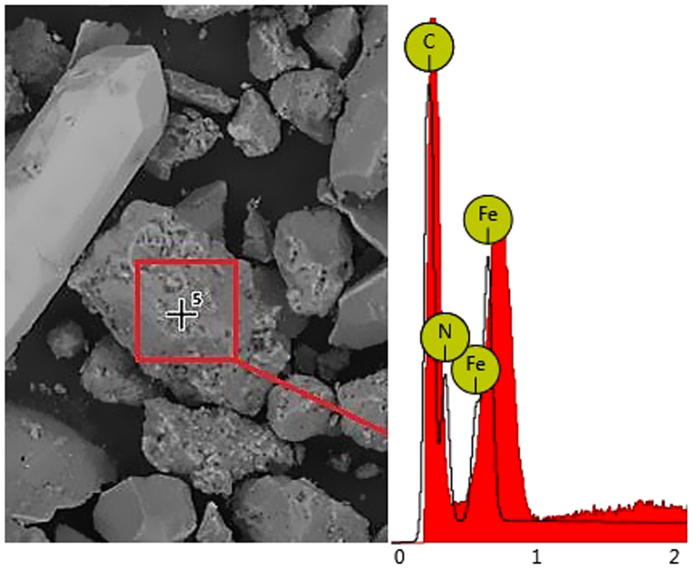
EDX scan and spectrum of the mine sand.

The erodent sand particles were collected from the town of Bandar Seri Iskandar located in Perak state of peninsular Malaysia. The composition of the sand samples was measured using EDX analysis. Figure 5 shows the EDX scan and spectrum of a rough sand sample, which was further refined for classification of the grain sizes. EDX analysis confirmed the presence of aluminium, silicon, phosphorus as well as carbon in the erodent sand sample. The existence of nitrogen, oxygen and fluorine in the sample was believed due to the surrounding air^6^,^7^.

**Figure 6:**
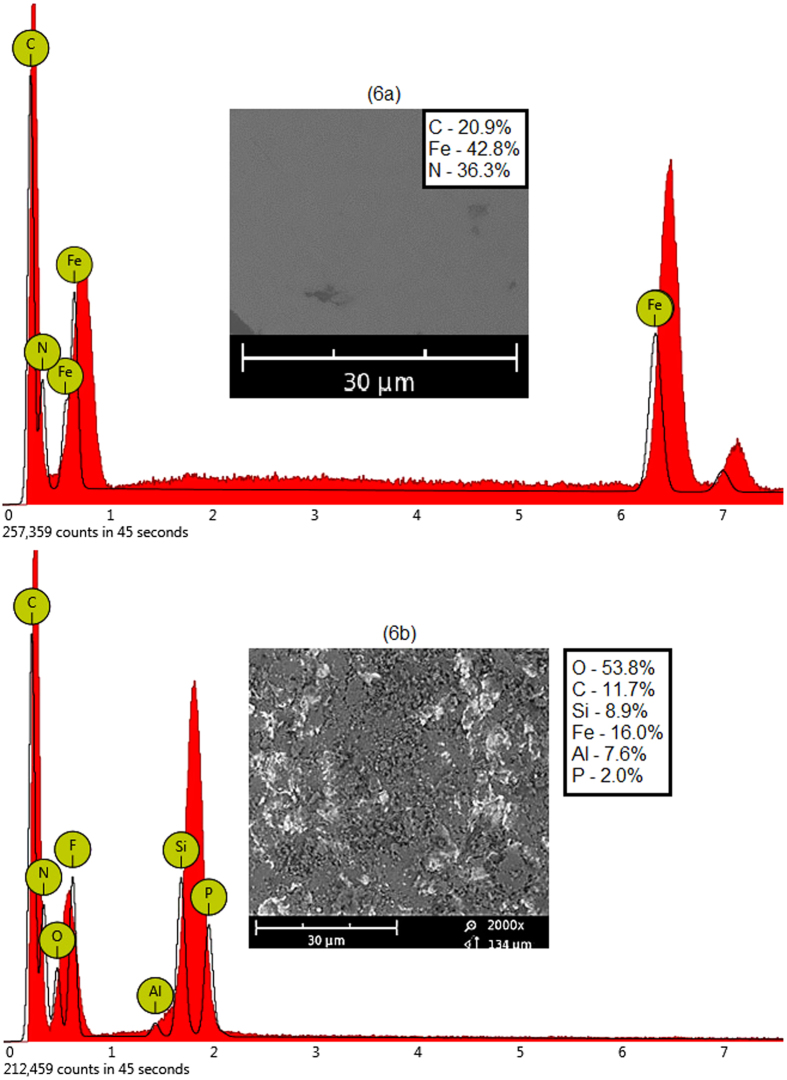
EDX of the mild steel (6a) untreated and (6b) treated.

**Figure 7:**
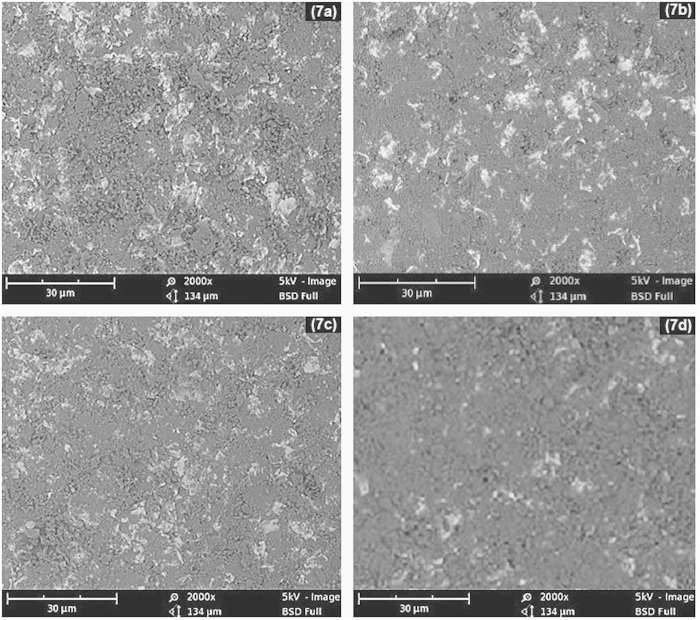
SEM images of the mild steel eroded with (7a) 150 μm particles at impact angle of 90°, (7b) 45 μm particles at impact angle of 90°, (7c) 150 μm particles at impact angle of 45° and (7d) 45 μm particles at impact angle of 45°.

The abrasive erosion investigations on mild steel performed using dry sand impact method under room temperature and atmospheric pressure conditions are reported in Figs 6 and 7. It was more likely a physical phenomenon rather than a chemical process. Figure 6 shows EDX spectra of untreated and treated mild steel coupons used as metal specimen in these experiments. Figure 6a reveals that the untreated sample was only composed of carbon, iron and nitrogen. Figure 6b shows EDX spectrum of the metal plate after erosion experiment. The composition of the eroded plate was significantly changed in these investigations. It reveals that some of the erodent particles were diffused into the metal surface during sand impact. The post-treatment metal composition was changed to carbon, silicon, ferum, aluminium and phosphorus. It was seen that the sand impact erosion also changes the composition of the metal samples^8^,^9^,^10^.

The past studies reported that the particle size significantly influences the metal surface erosion by determining the number of particles impacting the solid surface^7^,^8^,^9^,^10^. The particles below 10 microns are carried with the liquid medium and rarely impinge the wall, however, larger particles are likely to travel in the straight line and bounce off the walls. The larger particles with sizes above 1 mm move slowly and normally settle out of the fluid, therefore did not significantly harm the surface. There are also few evidences that the particles with sharp edges do more damages than those of rounded shapes^9^,^10^,^11^. However, it would be difficult to conclude on the effect of variability of the sharpness and hardness of the sand on the erosion rate in different production wells and fields^9^,^10^. Owing to the sharp surfaces, the sand particles used in the given work can be more lethal for the production pipe-lines.

SEM micrographs of mild steel samples bombarded for 1 hour with 45 μm and 150 μm sand particles at different impact angles are shown in Fig. 7. The untreated samples were polished coupons and almost free from scratches and rough patches, as shown in inset of Fig. 6a. After erosion experiments, the targeted regions of the samples were turned to dark circles revealing the high surface roughness. The degree of the surface roughness was greatly depended on the particle size and impact angle. A strong relationship was found between particle size and the damage scar size on the metal surface^9^,^10^. The coarse sand eroded the metal more adversely as compared to the fine particles.

It has been noticed that collision efficiency decreases with a decrease in particle size^10^,^11^,^12^. Quantitatively, the decrease in collision efficiency and consequently the erosion can be attributed to the lower inertia of the smaller particles which are not constrained to follow the air moving around a body on its way. Therefore the bigger particles will have higher inertia and momentum to impact the metal surface by causing significant erosion. However, the presented results prove that the fine particles can also cause significant erosion of the metal surface even if mass of the fines is smaller as compared to coarse sand. So the surface damages due to the fine particles can be significantly higher in the areas where coarse sand cannot reach in normal situations.

SEM images in Fig. 7 also reveal the effect of impact angle on surface damages. The highest metal erosion was noticed at the impact angle of 90°, which was gradually reduced by decreasing the impact angle to 30° in steps of 15°. At 90°, the accelerated particles were not exhibiting any angular slip and imparting maximum energy to the metal surface. However, at smaller impact angles, the sand particles exhibited an angular slip due to the tangential path created by the inclined metal surface. The particle impact force was split into axial and radial components. Herein, only the axial component was contributing to the energy transfer from sand particles to the metal surface. As a result of this, the sand impact was reduced and consequently the surface erosion.

**Figure 8:**
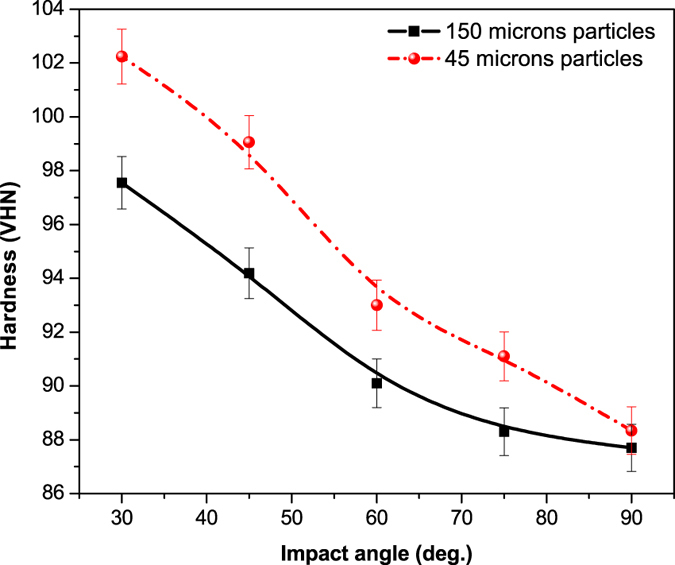
Micro-hardness of the steel samples eroded at different impact angles.

The micro-hardness of the treated steel samples as a function of impact angle is reported in Fig. 8. The hardness of the mild steel exhibited an increasing trend with impact angle. Overall hardness of the samples treated with 150 μm and 45 μm sand samples was found in the range of 87.7 to 97.55 VHN and 88.34 to 102.31 VHN, respectively. It reveals that the hardness and density of the sand particles impacting the metal surface were sufficiently higher to induce plastic deformation and evolution of the micro structures at the sample surface. The increased hardness of the samples treated with 45 μm shows that the smaller particles impact on the surface was forming the fine grains, marten sites and residual stress which subsequently increased the subsurface micro-hardness^13^.

In addition to the dry sand erosion, the aqueous erosion of the mild steel was also investigated using an electrochemical technique. LPR monitoring is an effective electrochemical technique, which is used to evaluate the erosion-corrosion of the metals. It involves the monitoring of a relationship between the current due to the electrons and the electrochemical potential. The corresponding LPR curves are used to measure the corrosion rates. In this relation, the polarization resistance varies inversely with the rate of corrosion. In this study, a triple electrode probe was added into the mild steel erosion process. The electrodes were electrically isolated from each other and from the process line^3^. A small potential (in the range of 20 mV) was applied between the electrodes without affecting the corrosion process. The corresponding current was monitored and measured. LPR which is the ratio of the applied potential and the current between the electrodes was measured for mild steel samples in slurries having 5% by weight of 45 μm and 150 μm sand particles.

**Figure 9:**
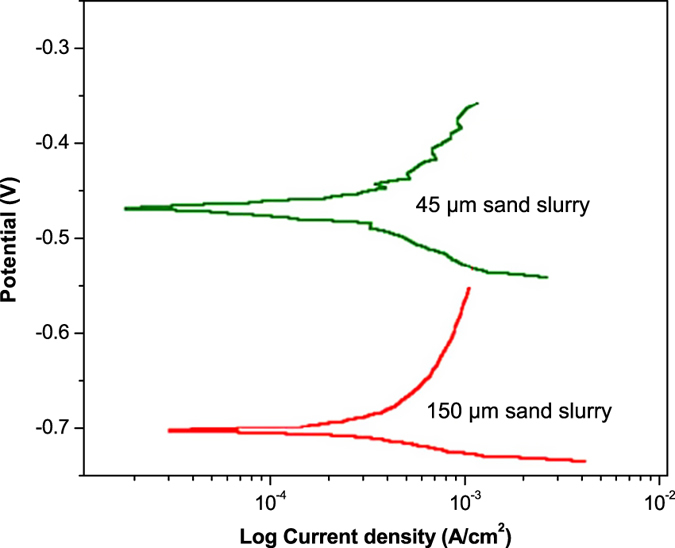
Polarization curves of mild steel treated with 45 μm and 150 μm sand slurries.

Figure 9 shows LPR curves of the mild steel corroded in slurries of 45 μm and 150 μm sand particles. The polarization behavior of the mild steel in 45 μm and 150 μm sand slurries was about 0.29 and 0.34 mm/year, respectively. These findings predicted high surface damages due to larger particles, which were expected in these experiments. Corrosion of the mild steel samples was explained in terms of polarization resistance (Ω.cm^2^) of the current-potential density curves. Herein, the polarization resistance of the samples was calculated using the equation^3^,^14^:


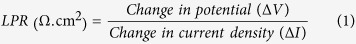


From this equation, the polarization resistance of the samples in 45 μm and 150 μm sand slurries was calculated about 949 Ω.cm^2^ and 809 Ω.cm^2^, respectively. A decrease in LPR of the samples generally suggests a decline in the polarization resistance in the presence of larger particles in the slurry. This trend reveals that the metal would suffer with higher corrosion rates in the fluids carrying the larger solid particles. The observed trend of polarization resistance can be clarified in terms of stability of the externally formed corrosion product layer. The LPR will remain more or less constant, if product layer is stable and is not separable from the metal surface. However, LPR value will fluctuate if the product layer is not stable. In this case, the layer forms and detaches again from the metal surface^14^.

## Conclusions

This study concludes that the density and sharpness of the sand particles, used in this study, were sufficiently higher to induce the deformation and evolution of the micro structures at the steel surface. The flow-lines will undergo higher corrosion rates in the presence of mechanically harder and larger particles in the fluid stream as compared to the relatively softer and smaller particles. Overall aqueous erosion of the mild steel due to electrochemical reaction remains relatively lower than the stream flow carrying the solid particles.

The sand carrying flow can deteriorate the metal surface fully or partly depending on the velocity, impact angle, mechanical strength and sharpness of the solid particles involved in the erosion process. Therefore in the absence of an accurate monitoring system or predictive model, the routine monitoring of the flow-line thickness would be required to warn about the erosion damages or impending loss of the containments. The sacrificial coupons can also be used to serve this purpose.

Although the anticipated metal erosion due to the larger particles was higher, the fine particles can also notably damage the metal surface. The hardness of the samples treated with smaller particles was found in close agreement with those treated with larger particles. However, this agreement can terminate with increase in exposure time by revealing high surface damages due to larger particles. Nevertheless, the surface damages can be appreciably reduced by changing the impact angle of the erodent particles with the metal surface^15^,^16^,^17^.

## Additional Information

**How to cite this article**: Naz, M. Y. *et al.* Electrochemical and Dry Sand Impact Erosion Studies on Carbon Steel. *Sci. Rep.* **5**, 16583; doi: 10.1038/srep16583 (2015).

## Figures and Tables

**Figure 1:**
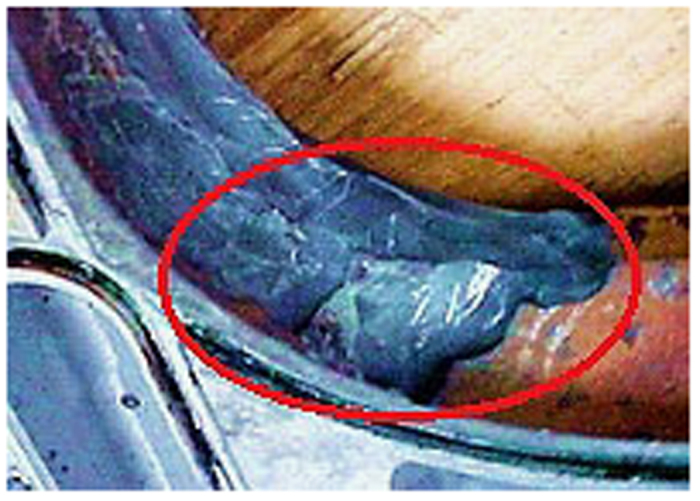
Failure caused by sand impact erosion of the mild steel.
